# Reply to Zachritz et al. Comment on “Petre et al. Tissue Bioconcentration Pattern and Biotransformation of Per-Fluorooctanoic Acid (PFOA) in *Cyprinus carpio* (European Carp)—An Extensive In Vivo Study. *Foods* 2023, *12*, 1423”

**DOI:** 10.3390/foods15071262

**Published:** 2026-04-07

**Authors:** Valentina Andreea Petre, Florentina Laura Chiriac, Irina Eugenia Lucaciu, Iuliana Paun, Florinela Pirvu, Vasile Ion Iancu, Laura Novac, Stefania Gheorghe

**Affiliations:** National Research and Development Institute for Industrial Ecology—ECOIND, Drumul Podu Dambovitei 57-73, Sector 6, 060652 Bucharest, Romania; petrevalentinaandreea@gmail.com (V.A.P.); irina.lucaciu@incdecoind.ro (I.E.L.); iuliana.paun@incdecoind.ro (I.P.); florinela.pirvu@incdecoind.ro (F.P.); vasile.iancu@incdecoind.ro (V.I.I.); laura.novac@ecoind.ro (L.N.); stefania.gheorghe@incdecoind.ro (S.G.)

We thank the correspondents [1] for their interest in our work [2] and for the opportunity to clarify analytical and interpretative aspects related to the occurrence of short-chain PFCAs (PFBA, PFPeA, PFHxA, and PFHpA) in tissues of PFOA-exposed common carp. We agree that rigorous source attribution is essential when discussing transformation mechanisms for PFAS. Below we address the specific points raised and summarize why, based on our QA/QC controls and the temporal behavior of the data, the reported tissue profiles are reliable and not attributable to dilution-water contamination.

## 1. Evidence That the Exposure Water Did Not Contain C4–C7 PFCAs During the Study

**Figure 1:**
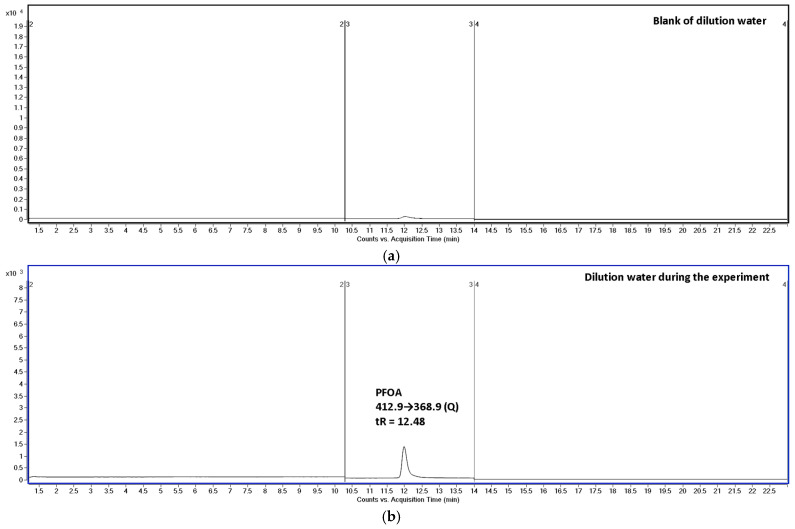
MRM chromatograms of dilution water: (**a**) blank of dilution water before the experiment and (**b**) dilution water during the exposure period. The PFOA peak (tR ≈ 12.48 min) was detected in the exposure water using the MRM transition 412.9→368.9 (Q), while no signals corresponding to short-chain PFCAs (PFBA, PFPeA, PFHxA, and PFHpA) were observed at their respective retention times.

In our experimental design, the dilution water was analyzed before the start of exposure and during the exposure period. In the pre-exposure dilution water, no PFAS analytes were detected under our LC–MS/MS conditions. During the exposure period, dilution-water analyses showed only PFOA, with concentrations remaining close to nominal values throughout (≥90%), while PFBA–PFHpA were not detected in dilution water at the relevant retention times (Figure 1). Importantly, we also performed dedicated PFOA stability experiments in water without fish (two aquaria, 10 and 100 µg/L), demonstrating that measured PFOA concentrations remained close to nominal over 96 h. Collectively, these controls support that (i) the aqueous exposure system did not generate detectable amounts of PFBA–PFHpA and (ii) the fish were exposed predominantly to PFOA in water.

Compound identification was based on retention time matching and MRM transition specificity using authentic standards analyzed under identical conditions. Full LC–MS/MS parameters (MRM transitions, collision energies, retention times) are provided in Table S3 (Supplementary Materials).

## 2. Tissue Occurrence of PFBA–PFHpA Is Supported by Chromatographic Confirmation and Matrix QA/QC

**Figure 2:**
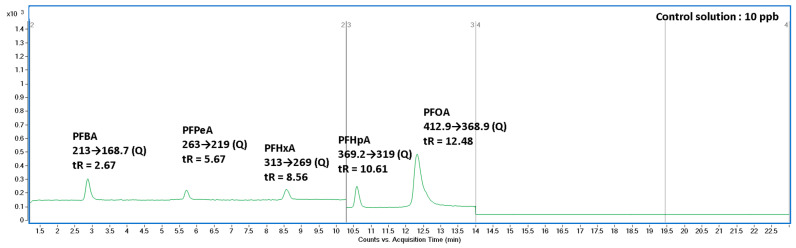
MRM chromatogram of a 10 ppb standard mixture containing PFBA, PFPeA, PFHxA, PFHpA, and PFOA. Each compound was identified based on its specific MRM transition and retention time.

All four short-chain PFCAs reported as transformation products were identified and quantified by targeted LC–MS/MS (MRM) using authentic standards (PFBA, PFPeA, PFHxA, and PFHpA) purchased from a reputable supplier and analyzed under the same instrumental conditions as tissue extracts. The method performance characteristics (recoveries, matrix effects, and LOQs for metabolites in fish matrices) were reported in the Supplementary Materials, and concentrations were background-corrected considering matrix effects and recoveries. We additionally compiled chromatographic evidence including: (i) dilution-water blanks (pre- and mid-experiment), (ii) a 10 ppb PFCA standard mixture (Figure 2), and (iii) representative chromatograms from organs where PFBA–PFHpA were observed. The absence of these analytes in the blank water combined with clear peaks in tissues at matching retention times supports that the reported signals represent true detections in biological matrices rather than general background contamination.

These standards were analyzed under identical LC–MS/MS conditions as the biological samples and were used for compound identification based on retention time matching and MRM transition specificity. Full analytical parameters are provided in Table S3 (Supplementary Information).

## 3. Time-Dependent Inverse Patterns Between PFOA and PFBA–PFHpA Support In Vivo Formation Consistent with Transformation

**Figure 3:**
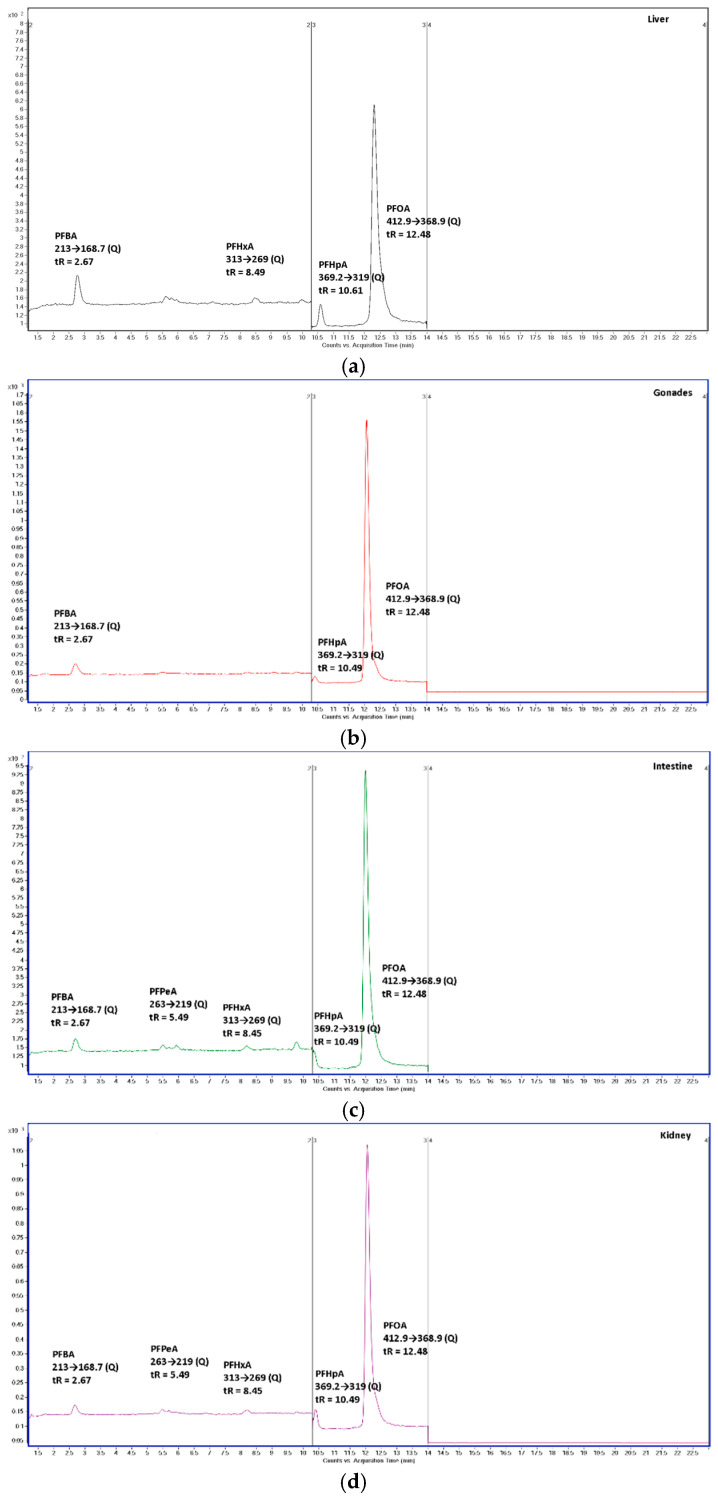

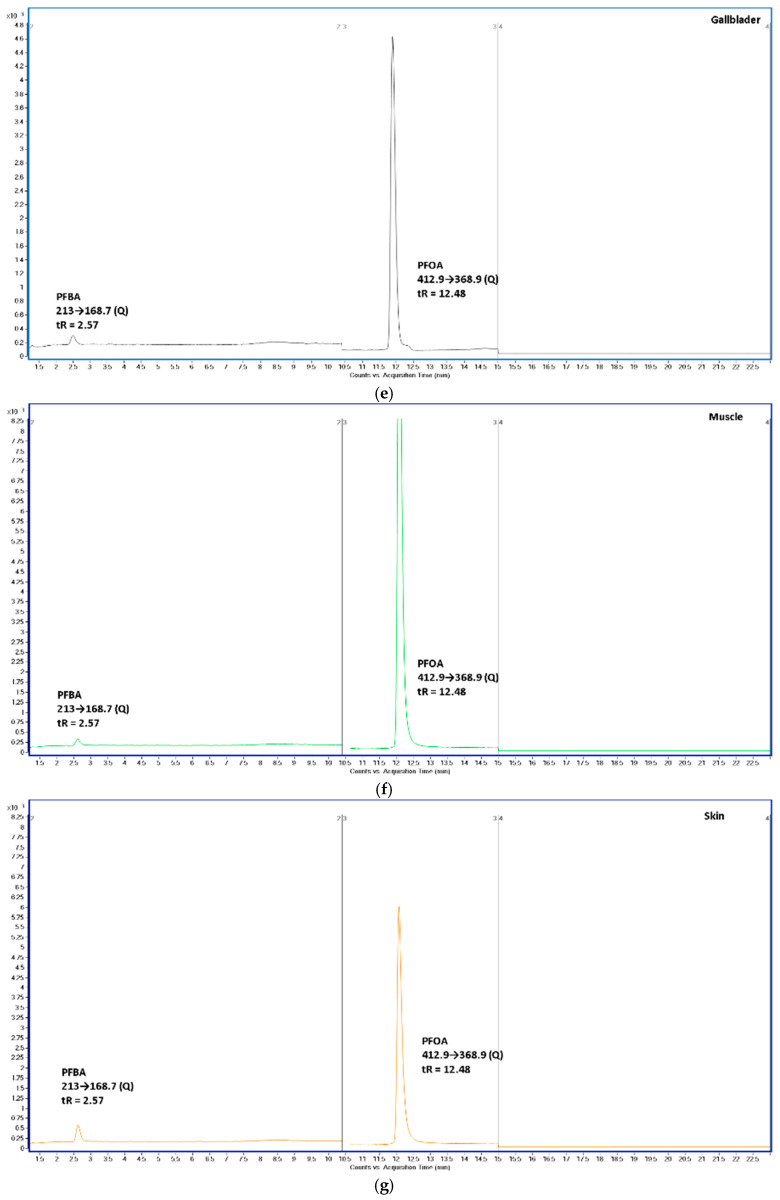
MRM chromatograms of PFOA and C4-C7 compounds in fish organs: (**a**) liver; (**b**) gonads; (**c**) intestine; (**d**) kidney (**e**) gall bladder; (**f**) muscle; (**g**) skin. Peaks were assigned based on retention time matching and specific MRM transitions (quantifier, Q) using authentic standards analyzed under identical LC–MS/MS conditions.

A key feature of our published dataset is the time-dependent behavior of PFOA and the short-chain PFCAs across the four sampling campaigns (8, 12, 14 weeks’ exposure; week 17 after depuration). As reported in the Results and Discussion, PFOA concentrations decreased or reached a plateau in multiple organs after the initial exposure stage (see Figure 1 and Figure 2 and the corresponding concentration tables in the Supplementary Materials: Tables S7 and S8). In parallel, PFBA–PFHpA were detected in organs with organ- and time-dependent profiles, with the overall evolution of metabolite concentrations shown in Figure 4, their distribution across organs shown in Figure 5, and their organ-specific evolution summarized in Figures S4 and S5 and Table S9. Importantly, we quantified the relationship between PFOA and the short-chain PFCAs using Spearman correlations (Tables S10–S19), which show (i) negative correlations between PFOA and one or more short-chain PFCAs in several organs and (ii) positive correlations among homologous PFCAs differing by one CF_2_ unit (PFHpA ↔ PFHxA ↔ PFPeA ↔ PFBA). We acknowledge that such correlations do not demonstrate causality and, therefore, cannot be considered definitive proof of biotransformation. Furthermore, similar patterns could arise from differential tissue-specific accumulation kinetics of co-occurring contaminants. However, this alternative explanation would require the presence of these compounds in the exposure system. Given that PFBA–PFHpA were not detected in dilution water during the experiment, the observed time-resolved and organ-specific patterns are more consistent with a linked, sequential process occurring in vivo during chronic exposure than with constant co-exposure from the aqueous phase. In support of these findings, selected representative chromatograms are provided below as illustrative examples (Figure 3).

Chromatograms are presented as representative examples to illustrate compound occurrence in biological matrices. Quantitative interpretation is based on integrated peak areas corrected for recovery and matrix effects (Table S4) and on the complete dataset reported in Table S9. Differences in signal intensity between panels reflect matrix-dependent responses and acquisition conditions and are not intended for direct quantitative comparison.

## 4. Regarding the Possibility of Homologous Impurities in the Dosing Material

We acknowledge that, in the absence of direct analysis of the dosing stock solution, the presence of trace homologous impurities (e.g., PFBA–PFHpA) cannot be entirely excluded. This represents a limitation when interpreting the mechanistic origin of the observed compounds. We also acknowledge that certain PFAS sources (notably ECF-derived materials) may contain homologous impurities. Our dilution-water monitoring during the exposure period consistently showed PFOA only, with no detection of PFBA–PFHpA. Thus, while we cannot entirely exclude trace impurities in the dosing stock without direct stock analysis, the available evidence indicates that co-exposure via the dilution water was not the driver of the observed tissue PFBA–PFHpA profile.

## 5. Clarification on Mechanistic Language and Limitations

We agree that isotopically labeled precursor experiments (e.g., ^13^C- or ^14^C-PFOA) provide the most direct provenance evidence. Such tracers were not available for our chronic study. Nonetheless, our conclusions are supported by: (i) verified absence of PFBA–PFHpA in dilution-water blanks and mid-exposure water, (ii) stable PFOA concentrations in water controls without fish, (iii) confirmed chromatographic detection of PFBA–PFHpA in multiple organs using authentic standards, and (iv) consistent time-dependent inverse relationships between PFOA and the short-chain PFCAs across sampling campaigns. Therefore, our published measurements represent true tissue concentrations under the tested conditions, and the data are consistent with in vivo formation of PFBA–PFHpA during chronic PFOA exposure.

In summary, our QA/QC controls demonstrate that PFBA–PFHpA were not present in dilution water (pre- and mid-exposure), and our LC–MS/MS data robustly confirm their occurrence in tissues with organ- and time-dependent patterns linked to PFOA dynamics. We welcome further work using isotopically labeled PFOA to provide definitive mass-balance provenance; however, the available evidence supports the validity of the reported tissue profiles and their interpretation as consistent with in vivo formation under our experimental conditions.
